# Age-Related Medicine

**DOI:** 10.3390/pharmaceutics11040172

**Published:** 2019-04-09

**Authors:** John Wahlich, Mine Orlu, Alpana Mair, Sven Stegemann, Diana van Riet-Nales

**Affiliations:** 1 Academy of Pharmaceutical Sciences, 4 Heydon Road, Great Chishill, Royston, Herts SG8 8SR, UK; 2 Department of Pharmaceutics, School of Pharmacy, University College London, 29-39 Brunswick Square, London WC1N 1AX, UK; m.orlu@ucl.ac.uk; 3 Effective Prescribing and Therapeutics, Health and Social Care Directorate, Scottish Government, Edinburgh EH6 5NL, Scotland, UK; Alpana.Mair@gov.scot; 4 Institute of Process and Particle Engineering, Graz University of Technology, Inffeldgasse 13, 8010 Graz, Austria; sven.stegemann@tugraz.at; 5 Medicines Evaluation Board in the Netherlands, Quality Department, Chemical Pharmaceutical Assessments, P.O. Box 8275, 3503 RG Utrecht, The Netherlands; da.v.riet@cbg-meb.nl

**Keywords:** polypharmacy, multiple morbidities, ageing, pharmaceutical sciences, product development, SIMPATHY

## Abstract

A meeting organised by the Academy of Pharmaceutical Sciences focussed on the challenges of developing medicines for older adults. International experts discussed the complexity introduced by polypharmacy and multiple morbidities and how the risk–benefit ratio of a medicine changes as an individual ages. The way in which regulatory authorities are encouraging the development of age-appropriate medicines was highlighted. Examples were provided of the difficulties faced by the older population with some medicinal products and suggestions given as to how the pharmaceutical scientist can build the requirements of the older population into their development of new medicines, as well as improvements to existing ones.

## 1. Introduction

The Academy of Pharmaceutical Sciences (APS, the professional body for pharmaceutical scientists in the UK) hosted a meeting on the challenges of age-related medicines as part of the APS@FIP Conference in Glasgow, UK, on 7 September 2018. Alpana Mair (Effective Prescribing and Therapeutics, Scottish Government), Sven Stegemann (University of Graz and Lonza) and Diana van Riet-Nales (Quality Department, Medicines Evaluation Board, the Netherlands) spoke at the meeting chaired by Mine Orlu (UCL School of Pharmacy). 

There is an increasing awareness that an individual’s response to disease and their ability to metabolise medicines changes with age. As a result, there have been a number of initiatives to encourage the development of medicines specifically for children. However, the changing world demographics due to increasing life expectancy [1], with increasing numbers of older people (the so-called ‘silver tsunami’), means that companies should start developing medicines designed for the specific needs that are prevalent in the older population. The APS meeting, organised by the APS Age-Related Medicines Focus Group, explored some of the current challenges and opportunities in developing these medicines. The meeting heard from some of the EU’s leading experts on the recent developments in this exciting area of the pharmaceutical sciences. 

## 2. What or Who is Considered as Old?

Discussing challenges of developing medicines for ‘older adults’ implies that the patient population of ‘old’ or ’older’ is clearly defined. The dictionary definition mentions concepts such as ‘having lived for a relatively long time’ or ‘belonging to a remote or former period in history’ [2]. On an individual basis, we might think ourselves that we are getting old when we need to take many medicines, start to make noise after any movement of our bodies or when we regard staying up to 11.30 pm as “living wildly”. 

This already suggests that it is very difficult to define ‘old’ in the context of human ageing. Chronological age, physical age and cognitive age may or may not be coincident. A number of different biomarkers, including telomere length or DNA/chromosomal damage, taken in combination, have been suggested as more objective measures to determine our actual biological age, but are measures requiring a lot of effort. In addition, assessment tools such as muscle mass or grip strength are easier measures and have been suggested to evaluate an individual’s functional ability [3]. Interestingly, people with a higher biological age at 38 years age faster than people with a lower biological age (i.e., the gap remains over their lifetime) [4]. 

Determining the age of an individual more objectively is an important aspect of healthcare provision. There is a good consensus today that lifestyle plays a key role in the ageing trajectory. In the Whitehall II cohort study, the investigators found a correlation between unhealthy lifestyles (low physical activity, smoking and alcohol consumption) in middle age and disability (defined as reporting difficulties with mobility and instrumental / basic activities of daily living) when people grew older (the odds ratio for 2–3 unhealthy lifestyles and disability was 2.69 (95% confidence interval = 2.26–3.19)) [5]. Surprisingly, there was no correlation between the low consumption of fruit and vegetables and disability.

Overall, increasing wealth and advances in medical and pharmaceutical sciences are contributing to more years of healthy life as well as longer life expectancy. A German study found that on average, today’s 75-year-olds are cognitively fitter and happier than the 75-year-olds of 20 years ago. These findings support statements that the rise in life expectancy is accompanied by an increase in years of health, at least for 60- to 80-years old (comparison of data from the Berlin Aging Study (BASE) collected in 1993 [6] and the repeat BASE II study in 2014 [7]). 

## 3. Multiple Morbidities

The huge differences in the health trajectories of individuals and their poor correlation with chronological age is a major challenge in considering someone ‘old’ from a healthcare perspective. There is evidence that the incidence of acquiring and accumulating chronic diseases increases with increasing age. A recent study showed that multimorbidity, defined by WHO [8] as the co-occurrence of two or more chronic medical conditions in one person, affects >30% of those 45–64 years old, rising to >65% in those 65–84 years old and >82% in those >85 years old [9]. In addition, the incidence appears to be increasing across all age groups beyond 18 years and is becoming more prevalent at an earlier age [10]. Barnett et al. have also reported that multimorbidities can occur 10–15 years earlier in individuals from the most deprived areas [11]. Multimorbidity is a worldwide phenomenon and is only loosely correlated with the per capita national gross domestic product of the country [12].

Older adults with multimorbidity experience a high symptom burden associated with increased healthcare utilisation, frequent visits to the emergency department, hospitalisation, admissions to nursing homes, and a reduced quality of life [13]. The authors used the Memorial Symptom Assessment Scale (MSAS) [14] to study community-dwelling individuals aged more than 74 years old in Sweden. They determined that pain was the symptom with the highest prevalence, frequency, severity, distress and symptom burden, with a lack of energy and dry mouth affecting half of the study group. In conclusion, they found that individuals with poor vision, a likelihood of depression and illnesses of the digestive system were most at risk of a high symptom burden and might need age-specific guidelines for appropriate medication management.

A study in the Netherlands and Belgium investigated the therapeutic burden and identified four ‘daily life domains’ with associated aspects that impacted patients with multiple morbidities [15]. These were: organisation of care (waiting and travelling times, attitude of healthcare practitioners (HCPs), etc.); medication (side effects, multiple medications and how to use them, absence of a review to determine if they are still relevant, etc.); patient’s role (accepting condition and treatment as routine, depression/hopelessness, resistance to visits by a doctor or to treatment, etc.); and impact on daily life (taking medicines when travelling, being on a special diet, limitations imposed by side effects, etc.). These findings highlight the importance of the disease and therapeutic burden on patient experience and perception.

Shippee et al. [16] had previously developed a functional, patient-centric model which addressed what they termed the ‘cumulative complexity’ faced by patients with multiple morbidities. This model described the balance between patient workload demands (related to the treatment, self-care and life in general) and the patient’s capacity to handle the demands (including functional morbidity, financial/social resources and literacy) as a result of the increasing disease and therapeutic burden. This model provides further evidence that a single disease–single medicine concept might not be applicable to multimorbid patients with polypharmacy. 

## 4. Polypharmacy

As the number of comorbidities increases, so does the number of medicines for the patient. Such multiple medication use may be referred to as polypharmacy. Many definitions of this exist: in some domains, polypharmacy is defined as the intake of more than one medicinal product when the medication burden exceeds a certain threshold; however, the most common definition is the intake of five or more medicines [17]. All this implies that the intake of two to four (or even more) medicines may be referred to as polypharmacy.

It is important to note that polypharmacy can be both appropriate and inappropriate. The label “inappropriate” applies when polypharmacy can be linked to a number of potentially undesirable effects:

- There is an increase in the patient’s frailty linked to the number of their medicines. Frailty increases by 1.5× with five or more medicines and 2× with 10 or more [18] (note that in this reference, the term hyperpolypharmacy is coined for ≥10 medicines).
- The patient’s adherence to taking their medicines declines with polypharmacy [19,20].
- The risk of adverse drug reactions (ADRs) increases with the number of medicines being taken in older patients, with the risk for severe ADRs also being linked to age and frailty [21]. Pimohamed et al. [22] surveyed the medicines and the classes thereof most likely to cause ADRs leading to hospital admissions. The top five (medicine/class and % of ADRs caused) were: non-steroidal anti-inflammatory drugs (NSAIDs) and aspirin (29.6%); diuretics (27.3%); warfarin (10.5%); angiotensin-converting enzyme (ACE) inhibitors (7.7%); and antidepressants (7.1%).

In order to better manage polypharmacy, Mair described the EU SIMPATHY (Stimulating Innovation Management of Polypharmacy and Adherence in The Elderly) consortium (of which the Scottish Government is the lead) and the Scottish Government’s approach to address appropriate polypharmacy. 

The SIMPATHY consortium was set up to explore how healthcare management programmes can be implemented to improve medication safety and prevent patient harm by addressing the appropriate use of multiple medicines (polypharmacy).

The results were published in guidelines on polypharmacy and how to reduce the adverse impacts [23,24]. The guidelines define appropriate and inappropriate polypharmacy (Table 1) and stress the importance of multidisciplinary healthcare provider teams working with the patients and the need to break down barriers between the healthcare professionals.

Mair described the seven-step plan to review the medications of a patient with polypharmacy to achieve appropriate prescribing as recommended in the guidelines. The plan takes a holistic view on the patient’s health situation and tries to balance the clinical needs and priorities, while at the same time using the patient’s wishes as the guiding objective (Table 2).

The importance of step 1, focussing on what the patient wants, was illustrated by an example where a patient with a history of cardiovascular disease, who was already taking a statin, ACE inhibitor, beta-blocker, a diuretic and paracetamol for multiple morbidities, then had gabapentin prescribed by their doctor without any discussion. The effect of this was to make the patient light-headed and they suffered a fall which hospitalised them and had a very negative effect on their quality of life. 

Another example was a patient who had poorly controlled asthma, but could not think about managing this until they had received treatment for the pain they were in from a separate morbidity. 

Necessary medicines, which are identified in step 2, might be those that have essential replacement functions (such as thyroxine) or prevent rapid symptomatic decline (e.g., medicines for Parkinson’s disease or heart failure). Unnecessary therapies might be those for temporary indications, with higher than usual maintenance doses or which have limited benefit and are evaluated in step 3.

The effectiveness of the existing therapy is evaluated in step 4. If the therapeutic objectives are not being achieved, the therapy can be re-evaluated and intensified with the existing medicine therapy or new risks to benefits of a specific medicine therapy analysed dependent on their morbidity.

The concept of ‘Number Needed to Treat’ (NNT), a measure of how many patients will benefit from the specific medication (calculated from the average number benefiting with a control in a clinical trial), can be used to assess the effectiveness of a drug therapy, including whether the specific patient will benefit from the therapy. A value of 1 indicates that all patients will benefit, while a higher value means less chance of achieving the desired outcomes [25].

A review of drug–disease and drug–drug interactions from published data and from observations by and answers to specific questions from the patient is evaluated in step 5. The guidelines also offer various charts summarising the risk of interactions. 

Part of this step also includes sick day review cards providing information to the patient as to which medicines they should stop taking if they are unwell (with vomiting/diarrhoea or fevers, sweats and shaking).

Step 6 considers the economic factors and whether there are alternative therapies, with an appropriate risk–benefit–convenience balance, that are more cost-effective.

In the final step, the patient’s willingness and ability to use the medicines is considered in order to assure the desired outcomes. Examples here might be the patient needing retraining on how to use their asthma inhaler, ensuring that the patient and their caregiver understand the outcome of the review, and/or determining whether the patient is willing and/or able to make the recommended lifestyle changes (e.g., giving up smoking). 

## 5. Regulatory Initiatives to Ensure Better Medicines for Older People

This section focusses on the activities of the European Medicines Agency (EMA). See [26,27,28] for further information.

Regulatory authorities have to balance the risks against the benefits for the putative new, generic or hybrid medicines based on the information with which they have been provided in the relevant application. Coupling this information with the Good Manufacturing Practice (GMP) certification of the manufacturing site to ensure the quality of the medicinal products allows them to grant a marketing authorisation (or variation) for the relevant products. The summary of product characteristics (SmPC), package leaflet (PL) and product label provide details of the patients for whom the medicine is intended based on clinical trials. The patient populations to which the medicine is being prescribed are often much wider in terms of age and multimorbidity than those studied in the clinical trials related to the application. 

Older adults might respond quite differently to the medication compared to younger adults in view of age-related changes in pharmacokinetics, pharmacodynamics and multiple morbidities [29]. Only for a few (mostly recently authorised) medicines is it stated that some of the products in the medicine portfolio are specifically intended for a specific subset of the population, e.g., children, adults with swallowing difficulties, etc.

Regulatory authorities have recognised that the needs of ‘special’ patient populations have to be considered during the medicine development process. Starting with the paediatric populations, older adults have been identified as another population requiring better tailored medicines. There are a number of similarities between paediatrics and the older population; for example, both have metabolisms and physiologies which differ from the majority of adults, both can have difficulty swallowing and both may require special dosing considerations. On the other hand, whereas the paediatric population and some of the older population have caregivers, a significant proportion of the older population may live independently and hence be responsible for their own medication management. The older population is also much more likely to have multiple morbidities and use polypharmacy. 

Recognition by the regulatory authorities led to the initiative to develop the EMA Geriatric Strategy [30,31] and to review the existing legislative framework with respect to the ageing population, as well as to “identify the gaps in regulatory and scientific knowledge and take appropriate measures to tackle them” [30] (note that there is no specific geriatric regulation). The measures have included the organisation of a workshop on geriatric medicine [32] and the provision of scientific advice during product development. In the latter case, the emphasis is on formulations and pharmaceutical product design which suit the needs of older adults to improve effectiveness. De Spiegeleer et al. [28] have suggested that the impact of current regulatory initiatives needs to be monitored, and if they are not successful in leading to better medicines for the older population, then the idea of geriatric investigation plans (GIPs; similar to paediatric investigation plans (PIPs)) may need to be considered. 

The regulatory initiatives also focus on the better representation of older adults as a targeted patient population. The ICH E7 [33] guideline provides directions on the inclusion of older adults in clinical studies to address their routine underrepresentation in clinical trials where the medicines are intended for the general population [31]. This guidance suggests that data should be presented for various age groups (for example, <65-, 65–74-, 75–84- and >85-year-olds) to assess the consistency of the treatment effect and safety profile for these patients with the randomised patient populations included in the major phase III clinical trials. EMA guidance on what defines frailty [34,35] emphasises that age is not the only consideration in assigning and assessing individuals in such trials, and frailty needs also to be taken into account. 

With the introduction of the ICH Q8 [36], covering pharmaceutical development, the definition of pharmaceutical quality was extended beyond the technical aspects by emphasising the requirement that “the product should be designed to meet patients’ needs and the intended product performance”. The needs of older adults and especially multimorbid patients with polypharmacy will almost certainly differ from those of younger patients. Therefore, more consideration has to be given during medicine development to the pharmacokinetic performance of the medicine, the ability of the patient to handle and use the medicine, and the medicine’s impact on the patient’s health and quality of life in situations of multiple morbidities and polypharmacy.

In accordance with the increasing patient focus, the EMA have recently published their draft reflection paper on the pharmaceutical development of medicines for use in the older population [37] (consultation closed 30 January 2018). Consistent with the adopted guidance for paediatrics, the document intends to summarise the existing data and knowledge on pharmaceutical medicinal product design for older adults. Information from the literature (e.g., [38,39]), post-marketing data, discussions with stakeholders on practical issues and a gap analysis on how existing marketing authorisations are not fully meeting the needs of older patients were reviewed. As a reflection paper, the document is not intended to provide guidance, but to provide useful information on developing medicinal products suitable for use in the older population and to promote discussion on the topics addressed [37]. 

In order to suitably acknowledge older people’s needs in pharmaceutical development, it is essential to respect that knowledge on the specific needs of older people may not be readily available among medicine developers in industry who are commonly trained in nonmedical sciences. Therefore, the draft paper includes an annex describing the general, sensory, motor and (patho-)physiologic issues prominent in older people and requiring specific attention in the (pharmaceutical) development of the medicine. For example, the older population may:

- experience difficulties in administering certain dosage forms (swallowing of tablets and capsules due to dysphagia, administering eye drops and patches, etc.);
- struggle to open certain packaging or dosage containers or handle dosing devices (due to poor handling capabilities);
- have higher risk for medication errors due to visual impairments (e.g., not being able to differentiate one white tablet from another) or therapeutic complexity;
- not be able to read and/or understand instructions and to appropriately follow these in daily life (due to poor eyesight, health literacy, cognitive abilities or lack of caregiver assistance).

The reflection paper also mentions the need to modify dosage forms to facilitate dosing through an enteral feeding tube or to fragment the medication to reduce the dosage or to mix with food to make it easier to swallow. However, all these modifications would need verification by industry before they can be included in the SmPC/PL; i.e., before information on these modifications becomes available to the user of the medicine. Validation might require longer in-use shelf lives to allow caregivers to prepare medicines to be left over weekends or holiday periods. Otherwise, these modifications are considered as being for off-label use, meaning they are the sole responsibility of the healthcare professional.

## 6. What Do Older Patients Expect from Pharmacotherapy?

Adlai Stevenson (an American politician) once said ‘in the end, it’s not the years in your life that count; it’s the life in your years’. Everyone’s personal goal should be to achieve ‘healthy ageing’, defined as “the process of developing and maintaining functional ability which enables wellbeing in old age” [8].However, there can be a mismatch between what healthcare professionals and caregivers regard as a healthy life (with a focus on body functioning, vital energy, resilience and care support) and what individuals regard as their ‘quality of life’, which also encompasses a positive life balance, future life perspective, social participation, active daily organisation, environmental support and relief from loneliness [40]. A focus purely on health matters can miss the attention needed to ensure an individual’s overall quality of life, especially as quality of life is becoming a more important goal for older patients. The importance of an individual’s quality of life is highlighted by data from Belgium on the demand for physician-assisted dying, which increased from 3.4% of the surveyed population in 2007 to 5.9% in 2013, with the proportion of requests granted rising from 1.9% to 4.6%. Among the reasons given were ‘low expected quality of life’ and ‘loss of dignity’ [41]. The issue of physician-assisted dying inevitably raises the question as to what constitutes ‘dead’, and the case of Jahi McMath in the US still being alive (albeit in a coma) after being declared dead by physicians is one of the most publicised cases on this matter [42].

Trewby et al. [43] highlighted the difference between a physician’s view on what constitutes a health benefit and a patient’s expectations in a study based upon a hypothetical cholesterol-lowering medicine. Three hundred subjects were studied using a written questionnaire and an interview. They were split into three groups consisting of those who had had just been discharged from a coronary care unit, those who were taking cardioprotective medicines but had no history of myocardial infarction, and those with no history and who were not taking cardioprotective medicines. The threshold of expected benefit versus the inconvenience of having to take a medicine long-term was 20%, 20% and 30% absolute risk reduction, respectively, for the three groups. These expected benefit figures need to be compared with the likely benefit, which is only <5% over a five-year period, even though it is statistically significant. The authors concluded that if the patient is told of the likely benefit, they may not be inclined to take the medicine. 

The complexity of interacting dosing regimens for an individual with multiple morbidities was illustrated for a patient suffering pain and taking three painkillers), a proton pump inhibitor (for stomach protection), Wobenzym (a combination of plant-derived enzymes, for joint swelling), a treatment for muscle cramps and prophylaxis for deep vein thrombosis. The dosing regimen necessary to comply with the various dosing schedules would require the patient to take one or more medicines at four different times in the morning, at three different times during each of the afternoon and evening and a final dose at 0300 h; a seemingly impossible regime to which to adhere.

Van Riet-Nales gave examples on how existing medicinal products are not meeting the needs of the older population. In work on the need to subdivide large tablets, which otherwise may be difficult to swallow, van Riet-Nales et al. [44] studied the use of three commercial tablet-splitter devices and the accuracy and precision with which they produced uniform tablet fragments when applied to 500 mg paracetamol tablets. Some tablet splitters gave very bad results, and subdivision by hand gave fragments which met the regulatory requirements best. The latter option may not be available to older persons as they may not have adequate dexterity (the results reported were achieved by a healthy 24-year-old). The authors concluded that HCPs need to appreciate that tablet division may lead to inaccurate dosing and that regulatory authorities should take appropriate actions to ensure the accuracy of splitter devices and, where feasible, reduce the need for their use in the first place. 

In a second example [45], individuals over 70 years old were interviewed about their medicines. Ninety-five percent reported problems and, in some cases, described the way in which they got around these issues (e.g., they might resort to using a sharp implement to open packaging or store medicines that look alike separately). While most of the issues were ones of inconvenience, 5% of the problems in combination with the patient’s management strategy were deemed sufficiently serious to cause moderate or severe clinical deterioration. 

Drumond et al. [39] conducted a literature review on the clinical evidence for the patient’s appropriateness, acceptability, usability or preference for pharmaceutical preparations. They summarised their findings and concluded that little attention is being given to these elements when designing a medicinal product. The implications, in particular where the patient is responsible for their own medication, are inappropriate use of the medicines or lack of adherence.

ICH Q8 [36] introduced the concept of the quality target product profile (QTPP) to define the requirements for a medicinal product during development. Stegemann [46] has highlighted the need to link this to the ‘real-world disease’ patient. In the case of the older population, factors such as their ability to handle the medicine packaging, cope with the dosing regimen (bearing in mind polypharmacy) and to distinguish one product from another should be taken into account. The advances in medicinal product design (such as controlled release products or requirements to take products before, with or after meals to optimise pharmacokinetics) might not be respected by patients due to prior learning. A patient simply sees a product that looks like one they have always taken and will use it and administer it in the same way. The consequence can be that they crush a controlled release product to make it easier to take, thereby destroying its release profile, or take all of their medicines at the same time as they are then more likely to remember to take them.

The pharmaceutical scientist needs to consider how the product they are developing will be used. They need not only to work to a QTPP, but also to a ‘targeted patient profile’. It may not be necessary to tailor the product to a particular age group, as with some thought, they could evolve ‘traditional design’ to ‘universal design’ [46]. For example, the round screw cap on medication bottles could be replaced by a triangular one to make it easier to be opened by patients with a poor grip. Likewise, tablet appearance could be improved by using high-contrast imprints rather than embossing to allow identification by patients with poor eyesight. A recently published book, ‘Developing Drug Products in an Aging Society: From Concept to Prescribing’ [47], provides further practical guidance. 

In a similar message to that given by Mair on improving polypharmacy, Stegemann [46] suggests that patient-centric medicinal products can be achieved by interdisciplinary collaboration between the clinical, pharmaceutical, regulatory and marketing experts. This would obviously have input from inclusion of the older population in the clinical trials and involve listening to their experiences using the medicinal products. The same message is given in the (draft) EMA reflection paper.

## 7. Conclusions

Life expectancy is increasing, and even though chronological age does not necessarily correlate with ill health, the incidence of multiple morbidities, which can start in early adulthood, increases with age. The availability of new medicine therapies plays an important role in an individual achieving healthy ageing and maintaining a good quality of life, as well as addressing the impact of an ageing population on the demands of caregivers and society overall. However, the challenges of polypharmacy, medicine adherence and the suitability of medicines for the older population need to be tackled. A holistic approach where academia, industry, HCPs and patients work together to understand the patient’s needs and abilities and how they can best be addressed in the provision of appropriate medication is essential. Especially, the pharmaceutical scientist has an important role to ensure that medicinal products, delivery systems and dosing regimens meet the patient’s and caregiver’s requirements and do not just add to their burden. Clinicians should realize that lack of efficacy or side effects may not relate to the medicine itself, but rather to the dosage form, formulation/strength and/or the selected trademark, as patients may be unable to use certain products as intended. 

## Figures and Tables

**Table 1:** Criteria for appropriate and inappropriate polypharmacy.

Appropriate Polypharmacy	Inappropriate Polypharmacy
All medicines are prescribed for the purpose of achieving specific therapeutic objectives that have been agreed with the patient.	There is no evidence-based indication, the indication has ended, or the dose is unnecessarily high.
Therapeutic objectives are being achieved or there is a reasonable chance they will be achieved in the future.	One or more medicines fail to achieve the therapeutic objectives they are intended to achieve.
Medicine therapy has been optimised to minimise the risk of ADRs.	One or the combination of several medicines cause unacceptable ADRs or put the patient at an unacceptably high risk of such ADRs.
The patient is motivated and able to take all medicines as intended.	The patient is not willing or able to take one or more medicines as intended.

**Table 2:** Seven-step plan for polypharmacy medicine review as detailed in the SIMPATHY guidelines.

Step	Objective	Action
1	Aims	Identify the objectives based on what matters to the patient
2	Need	Identify necessary medicine therapies
3	Need	Identify unnecessary medicine therapies
4	Effectiveness	Are the therapeutic objectives being achieved?
5	Safety	Does the patient have or are they at risk of ADRs?
6	Cost-effectiveness	Is the medicine therapy cost-effective?
7	Patient-centric consideration	Is the patient willing and able to take the medicine therapy as intended?
